# Anatomically corrected malposition of great arteries

**DOI:** 10.4103/0974-2069.74057

**Published:** 2010

**Authors:** Anuradha Sridhar, Raghavan Subramanyan, Sudeep Verma, Smartin Abraham

**Affiliations:** Department of Pediatric Cardiology, Frontier Life Line Hospital, Dr. K.M. Cherian Heart Foundation, Chennai, Tamil Nadu, India; 1 Department of Pediatric Cardiac Surgery, Frontier Life Line Hospital, Dr. K.M. Cherian Heart Foundation, Chennai, Tamil Nadu, India

**Keywords:** Abnormal spatial relationship, anatomically corrected malposition of great arteries, malposed arteries

## Abstract

Anatomically corrected malposition of great arteries (ACMGA) is a rare form of congenital heart disease in which the great arteries arise above the anatomically correct ventricles but have abnormal spatial relationship. We report the case of a 26-year-old female with ACMGA and tunnel type of subaortic obstruction. The abnormal relationship and segmental arrangement necessitates systematic approach in evaluation for proper diagnosis and surgical repair. This unusual case is reported for its rarity and to highlight the need for awareness to differentiate it from other more common conditions.

## INTRODUCTION

Anatomically corrected malposition of great arteries (ACMGA) is a condition in which the great arteries are abnormally related to each other and to the ventricles but arise from the anatomically correct ventricle. The abnormal relationship and segmental arrangement necessitates a systematic approach in evaluation for proper diagnosis and surgical repair.

## CASE REPORT

A 26-year-old female with NYHA class III dyspnea on exertion and easy fatigability was referred for surgery with the diagnosis of congenitally corrected transposition of great arteries (CCTGA), restrictive subaortic ventricular septal defect (VSD) and severe subpulmonic left ventricular outflow tract (LVOT) obstruction. She was on Propranolol. Clinical examination showed an acyanotic patient with low volume pulses and a grade 3/6 ejection systolic murmur over the left sternal border. Chest X-ray revealed normal heart size, left ventricular (LV) type of apex, right aortic arch, and convex left upper border, suggestive of dilated ascending aorta and normal lung vascularity.

Transthoracic echocardiography (TTE) showed situs solitus, levocardia, AV concordance, side by side orientation of the ventricles, and an anterior leftward aorta. The aorta appeared to override the interventricular septum but no definite VSD could be seen probably because of poor echo window. The pulmonary artery was seen to arise from the right ventricle with no evidence of pulmonary stenosis. A high velocity jet was seen in the subaortic region with a gradient of 100 mmHg. Double outlet right ventricle (DORV) with severely restrictive VSD, L-malposed aorta and unrestricted pulmonary blood flow was suspected on echo.

**Figure 1:**
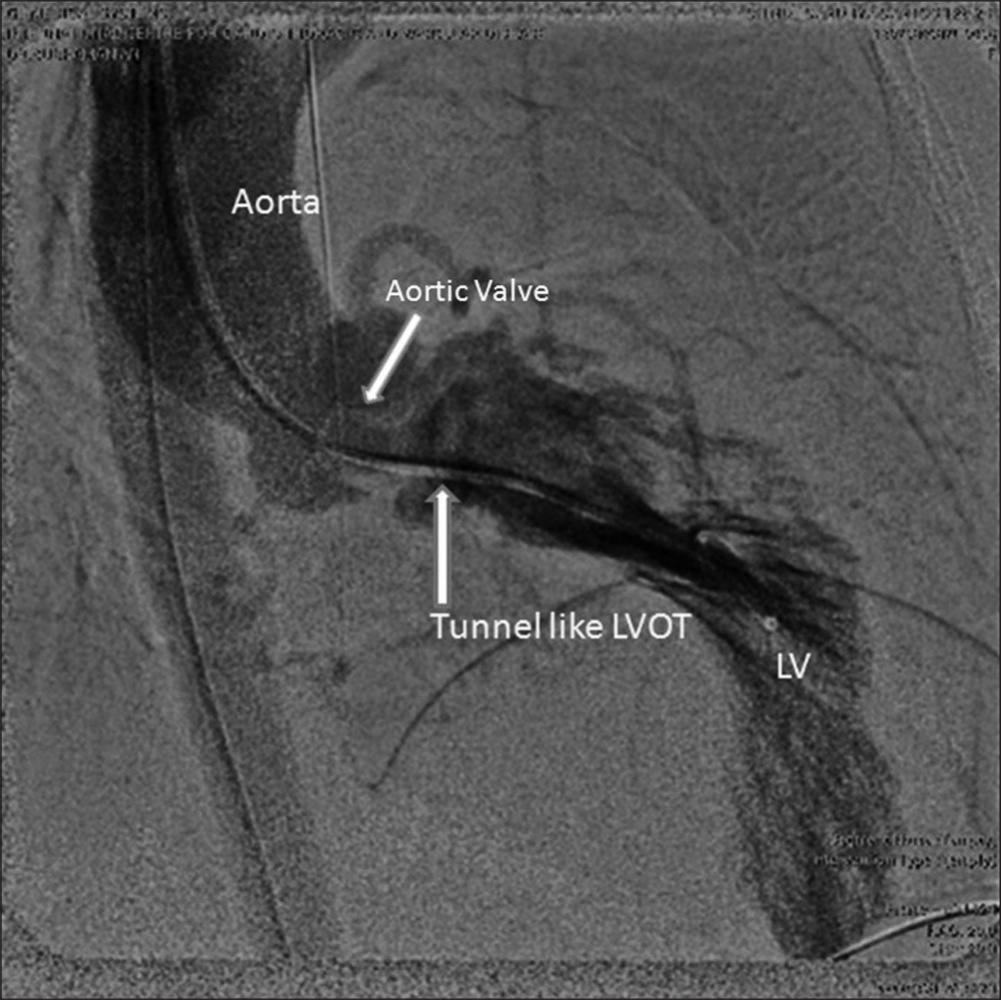
LV angiogram in frontal view with digital subtraction showing hypertrophied LV with elongated and narrowed LVOT. Systolic narrowing was also noted in the mid-LV cavity

**Figure 2:**
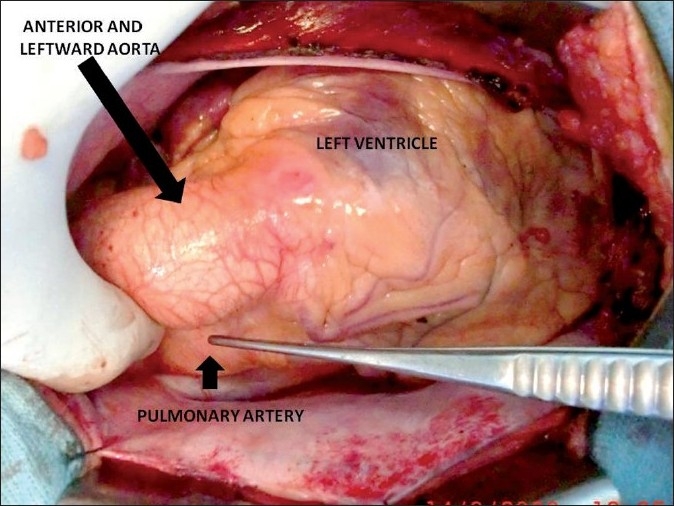
Surgical photograph showing the leftward and anterior aorta

**Figure 3:**
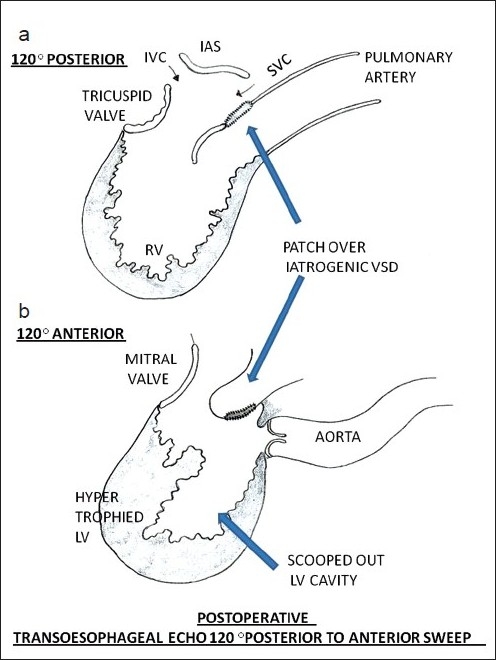
Sketch of the transesophageal views (a) 120° posterior tilt and (b) 120° anterior tilt of relationship of great arteries and ventricles

Electrocardiogram (ECG) revealed sinus rhythm, right axis deviation and LV dominance. The right axis deviation in the ECG was probably due to the side by side orientation of the ventricles. Catheterization showed situs solitus, atrioventricular (AV) and ventriculoarterial (VA) concordance with L-malposed aorta, LV pressure of 215 mmHg and RV pressure of 25 mmHg. There was a gradient of 140 mmHg during pullback from LV to aorta, suggesting severe subaortic LVOT obstruction. LV angiogram [Figure 1] showed severe hypertrophy of LV, long tunnel-like fixed subaortic stenosis and mid-ventricular systolic narrowing. A diagnosis of ACMGA with severe tunnel-like obstruction of the LVOT was made after catheterization. She underwent LVOT resection and LVOT enlargement with modified Konno technique (ventricular septum opened below the subaortic area with resection of hypertrophied septum, followed by closure of iatrogenic VSD with bovine pericardial patch). The abnormal segmental relationship [Figure 2] necessitated a combined transaortic and transatrial approach. She had an uneventful postoperative period. Postoperative echocardiogram revealed good relief of LVOT obstruction with residual gradient of 45 mmHg, mainly at mid cavity region [Figure 3]. On 6 months follow up, she was asymptomatic with mild to moderate AR and LVOT turbulence with a gradient of 20 mmHg.

## DISCUSSION

ACMGA, a rare condition, was first characterized by Van Praagh *et al*. in 1975.[1,2] There are four types of ACMGA: Type 1 (S,D,L) – situs solitus, d-loop ventricles, left and anterior aorta; Type 2 (S,L,D) – situs solitus, l-loop ventricles, right and anterior aorta; Type 3 (I,L,D) – situs inversus, l-loop ventricles, right and anterior aorta; and Type 4 (I,D,L) – situs inversus, d-loop ventricles, left and anterior aorta. Types 1 and 3 are physiologically corrected, whereas Types 2 and 4 have transposition physiology. The associated anomalies are VSD, right ventricular outflow tract (RVOT) obstruction, subaortic obstruction, right ventricular hypoplasia, juxtaposed atrial appendage and right aortic arch. Despite VA concordance, the aorta is supported by a muscular subaortic infundibulum which can be obstructive. This is due to the posteriorly placed LV connecting to an anterior aorta. Though systemic outflow tract obstruction is considered a commonly associated anomaly in ACMGA, there are a very few cases reported.[3,4] The embryological basis for this condition is described by Goor and Edwards[5] as isolated inversion of conus which implies inversion of conal musculature together with the arteries in reverse direction to that normally seen. The clinical presentation and findings in these patients depend upon the type and severity of associated anomalies. The elongated and unusual horizontal course of the LVOT in an adult patient with poor acoustic window made diagnosis by transthoracic echo difficult, and a wrong diagnosis of restrictive VSD was made. Transesophageal echo, computed tomography (CT) angiography, magnetic resonance angiography are additional noninvasive modalities of investigations which can be used to assess the VA connections and the spatial relationship of great arteries. Cardiac catheterization is indicated if there is need for hemodynamic assessment before surgery.

Surgical results have been good (92% survival) in those with situs solitus and AV concordance (S,D,L). However, when there is either AV discordance or hypoplastic right heart structures, or both conditions, the outcome after palliative procedure has been poor (29% survival).[6]

ACMGA is a rare entity but needs to be considered while differentiating congenital cardiac conditions with malposition of great arteries. The diagnosis becomes difficult in grown up patients with poor echo window. These patients can easily be misdiagnosed to have common conditions like CCTGA, DORV or transposition of great arteries (TGA) which are associated with malposition of great arteries. A systematic approach in preoperative evaluation will help in avoiding misdiagnosis of this rare condition. Accurate preoperative diagnosis and thorough assessment of the hemodynamics are essential for successful surgical outcome.
